# The Role of Adjuvant Single Postoperative Instillation of Gemcitabine for Non-Muscle-Invasive Bladder Cancer: A Systematic Review and Meta-Analysis

**DOI:** 10.3390/diagnostics12051154

**Published:** 2022-05-06

**Authors:** Georgios Koimtzis, Vyron Alexandrou, Christopher G. Chalklin, Eliot Carrington-Windo, Mark Ramsden, Nikolaos Karakasis, Kit W. Lam, Georgios Tsakaldimis

**Affiliations:** 1 Cardiff Transplant Unit, University Hospital of Wales, Cardiff and Vale University Health Board, Cardiff CF14 4XW, UK; chris.chalklin@gmail.com (C.G.C.); ecarrington-windo@outlook.com (E.C.-W.); markramsden@hotmail.com (M.R.); kit.lam@wales.nhs.uk (K.W.L.); 2 Urology Department, General Hospital of Thessaloniki “G. Gennimata-Agios Dimitrios”, Elenis Zografou 2, 546 34 Thessaloniki, Greece; vyrwnal@hotmail.com (V.A.); karakasnik@gmail.com (N.K.); g.tsakaldimis@hotmail.com (G.T.); 3 Department of Medicine, Democritus University of Thrace, Administrative Building, 6th km, 681 00 Alexandroupoli, Greece

**Keywords:** bladder cancer, intravesical chemotherapy, gemcitabine

## Abstract

Bladder cancer is a heterogeneous disease with variable natural history. Non-muscle-invasive bladder cancer has a favorable prognosis following transurethral resection, but the optimal adjuvant chemotherapy plan is still in debate. The aim of this study was to evaluate the effect of the adjuvant intravesical administration of a single dose of gemcitabine in the outcome of this disease. For that purpose, we performed a systematic review and meta-analysis on available randomized control trials on MEDLINE, EMBASE, Cochrane, Scopus, and Google Scholar databases. Ultimately, two studies were included with a total number of 654 patients. The statistical analysis performed showed that a single post-operative intravesical dose of gemcitabine does not affect the recurrence rate of non-muscle-invasive bladder cancer compared to placebo. Therefore, this therapeutic strategy does not offer any significant improvement on the outcomes of the disease. Nonetheless, due to the plethora of available therapeutic agents and treatment strategies, further research is needed to establish the optimal treatment in this category of patients.

## 1. Introduction

Bladder cancer (BC) is a highly heterogeneous disease with a variable natural history [1,2]. It can range from low-grade Ta tumors that progress slowly and only require endoscopic treatment and surveillance without posing an actual threat to the patient, to high-grade tumors with a very high malignant potential that can lead to significant progression and high cancer death rates [1,2]. BC is the 4th most common cancer in men and the 13th most common in women in the western world and the second most common genitourinary malignancy [1,2,3]. More specifically, in the United Kingdom (UK), it is the seventh most common cancer overall, with 10,399 cases and 5081 deaths in 2011 [2]. In the United States of America (USA), BC is the sixth most common malignancy and comprises 5% of new cancer diagnoses [4]. In 2018, it was estimated that 700,000 patients were living with the disease in the USA [3].

BC affects males three to four times more commonly than females [2,4,5], making it the eighth most common cause of cancer death [6,7,8]. At the same time, however, female patients with BC have more advanced tumors at the time of diagnosis [6,9], while female gender has also been associated with a higher risk of disease recurrence, progression, and death rate following treatment [10,11,12,13,14,15,16,17,18,19,20,21,22,23,24,25,26]. BC is also more common in people of White ethnic background than it is in people of Afro-American or Hispanic origin [4,5]. However, there are also many other risk factors involved. Advanced age plays an important role, as the majority of patients are diagnosed above the age of 55 with a median age of diagnosis between 65 to 70 years [1,2,4]. Nonetheless, the most important modifiable risk factor for BC is cigarette smoking, as it seems to be responsible for approximately two-thirds of BC in men and one-third in women and has been estimated to be a contributing factor in up to 50% of all cases in the USA [5,27,28]. This is a result of the carcinogenic effect of tobacco compounds such as aromatic amines and N-nitroso compounds, which cause DNA damage in the form of base modifications, double-stranded breaks, and bulky adduct formation [29,30]. Opium smoking has also been associated with BC [29,31]. Another significant risk factor for BC is occupational carcinogen exposure, which accounts for 5–6% of the overall attributable risk of BC [29,32]. There is also inference that metabolic syndrome could be a risk factor given altered lipid metabolism in most cancers. This corresponds with biomarkers such as pseudocholinesterase activity and ratio of triglycerides to HDL cholesterol in having the potential to serve as a biomarker for bladder cancer [33]. According to a large number of studies, the highest increase in the relative risk for BC was observed among tobacco, dye, and metal workers [29]. Specifically, agents with a suspected or established role as an occupational bladder carcinogen include 4-aminobiphenyl, 2-naphthylamine, 4,40-methylenebis(2-chloroaniline), toluene, metal working fluids, perchloroethylene, polyaromatic hydrocarbons (PAH), and diesel exhaust [29,34,35]. Other important risk factors include dietary factors, such as vitamin D deficiency [36] and consumption of animal protein and processed meats [37,38]. Finally, environmental carcinogens that have been associated with an increased risk for BC include arsenic [39,40,41] and nuclear power plant and shale gas extraction [42,43]. Lifestyle factors can also have a protective effect. Encouraging exercise and a diet rich in fruit and vegetables can reduce one’s risk of bladder cancer. As such, epidemiological and pre-clinical studies have correlated flavonols (kaempferol, myricetin, and fisetin) with a reduction in genito-urinaray malignancies in epidemiological studies [44].

The vast majority of BC originates from urothelial, or ‘umbrella’, cells that cover the lumen of the urinary bladder [45,46]. BC can be categorized in many ways [46]. Tumor stage is assigned by evaluating the depth of bladder wall invasion. BC that is confined to the urothelium (stage Ta) and to the lamina propria (stage T1) are defined as non-muscle-invasive BC (NMIBC) and are approached and managed differently from tumors that have grown beyond that level (stage T2 and above defined as muscle-invading BC, MIBC) [46]. NIMBC comprises up to 75% of the cases of BC [2,47,48]. The American Urological Association (AUA) stratifies patients with NMIBC into three categories: low, intermediate, and high risk [46]. The prognosis of NMIBC is generally good with 43% and 33% 5-year recurrence-free survival rates for patients with low and intermediate risk NMIBC, respectively [46], but up to 21% of patients with high-risk NMIBC will progress to MIBC [2,49,50]. The most common clinical presentation of BC is macroscopic hematuria [46]. However, BC can also manifest itself clinically as isolated microscopic hematuria (urinalysis revealing three or more erythrocytes per high-power field), symptoms of bladder irritation on voiding, or even as an incidental tumor identified on imaging performed for other diagnostic purposes. The diagnostic approach includes cystoscopy and endoscopic resection, imaging such as computed tomography (CT), or magnetic resonance imaging (MRI) urogram, mainly to assess the upper urinary tract and the use of urine tests and biomarkers [46].

NMIBC is mainly treated by transurethral resection (TUR) [51,52], but the high risk of recurrence and disease progression in a high percentage of patients necessitates further treatment with intravesical instillation of chemotherapy or immunotherapy [46,52]. For that purpose, there is currently a large variety of immunotherapeutic and chemotherapeutic agents that includes bacille Calmette–Guérin (BCG), mitomycin C, doxurubicin, epirubicin, and valrubicin [53,54]. Mitomycin C has traditionally been the drug of choice for this purpose, but it has been associated with irritative voiding symptoms and occasional bladder necrosis [55]. Moreover, available evidence on optimal dose and duration of intravesical chemotherapies other than BCG is limited [53].

Gemcitabine is a cell-cycle phase-specific chemotherapeutic agent (pyrimidine antimetabolite) that has been brought to light recently. The mechanism of action of gemcitabine primarily involves killing cells that undergo deoxyribonucleic acid (DNA) synthesis (S-phase), while it also inhibits the progression of cells through the G1/S-phase boundary [56]. Gemcitabine is metabolized inside the cytosolium to its active diphosphate and triphosphate metabolites. The cytotoxic outcome of gemcitabine is ultimately the result of the joint action of the diphosphate and the triphosphate nucleotides that leads to inhibition of the synthesis of DNA [56]. Gemcitabine has been proven to have biological activities in a wide range of solid tumors, such as pancreatic neoplasms, biliary tract cancers, sarcomas, non-small cell lung cancer (NSCLC), malignant pleural mesothelioma, breast cancer, and nasopharyngeal carcinoma [55,56]. Currently, gemcitabine seems to hold the greatest potential in becoming the chemotherapeutic agent of choice for intravesical therapies on the prevention of recurrence and progression of NMBIC [57].

The aim of this systematic review and meta-analysis was to evaluate the effect of adjuvant single postoperative instillation of gemcitabine on recurrence of NMIBC.

## 2. Materials and Methods

This study is a systematic review and meta-analysis that was carried out without a pre-existing registered protocol. A systematic and detailed electronic search of the literature was performed to identify randomized control trials (RCTs) on the outcomes of single adjuvant intravesical instillation of gemcitabine for NMIBC. The MEDLINE and EMBASE databases were searched from 1 January 2006 until 30 November 2021. Studies before this date were not included as they were considered to have been outdated and superseded by previous research. The following MESH terms, keywords, and search strategy were used for the MEDLINE database:

(“bladder”[All Fields] AND “neoplasms”[MeSH Terms] AND “gemcitabine”[Title]) AND (randomizedcontrolledtrial[Filter]).

Additional search was performed using similar search strategies in the Cochrane, Scopus, and Google Scholar databases. Finally, search for grey literature was conducted on the websites of international urological associations and networks and on available data from urological conferences.

Two independent reviewers (K.L. and M.R.) performed the original literature search on the online databases and then evaluated the retrieved articles for their relevance. Initially, articles were screened according to their title, and duplicate records were removed. Afterwards, titles and abstracts were examined for their relevance, and any non-relevant papers were excluded. Ultimately, full-text copies of the remaining articles were obtained for further eligibility screening and subsequently for data extraction and inclusion in the qualitative and quantitative analysis. In cases of disagreement between reviewers, a third independent reviewer (E.C.W.) was involved and, ultimately, either a consensus was reached, or the majority opinion was used for the final decision. Inclusion criteria were: (1) randomized control trials; (2) publication date after January 2006; (3) English language; (4) papers involving only adults; (5) animal models excluded; (6) single adjuvant intravesical instillation of gemcitabine used for NMIBC and compared to placebo; and (7) data on recurrence rate and/or survival rate and hazard ratio with 95% confidence intervals (CI) present. Studies that did not meet the above-mentioned criteria were excluded. Each article was studied independently for data extraction.

Data extracted from each article selected for analysis included the number of patients in each study and the participants in each arm (control and intervention), patients’ demographics (age and sex), the number of tumors identified at the time of index TUR, the tumor type (primary or recurrent), gemcitabine dosage and administration method, time of follow-up, time of recurrence, and hazard ratio with 95% CIs.

Data extracted were used in this meta-analysis to calculate the pooled hazard ratio and form the respective forest plot comparing the effect of gemcitabine administration in NMIBC recurrence rate to placebo, using a random-effect model. The assumption of data homogeneity is proven with the use of the chi-squared test and the calculation of the I^2^ index. Data in this study are presented as mean ± standard deviation. All the statistical analyses were carried out using Reviewer Manager 5.4.1 software (Review Manager (RevMan) [Computer Program]. Version 5.4.1, Copenhagen: The Nordic Cochrane Centre, Denmark, The Cochrane Collaboration, 2020). In the current study, the value of *p* < 0.05 was used as the level of statistical significance.

This systematic review was prepared following the PRISMA checklist.

## 3. Results

**Figure 1:**
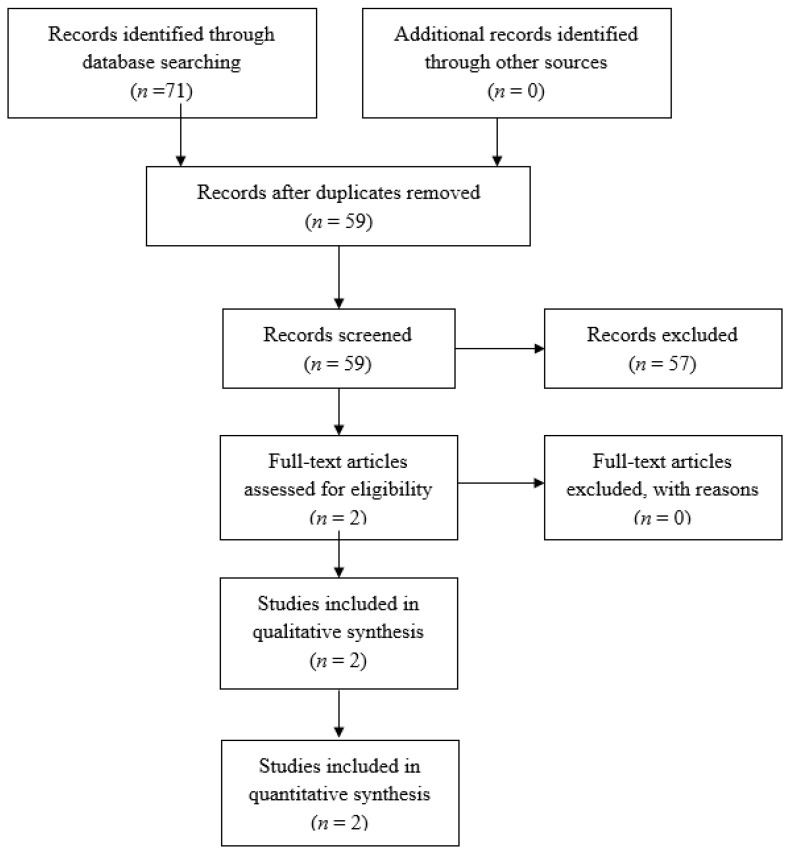
Flowchart depicting the selection process for inclusion of manuscripts in the study.

The initial search of the electronic databases resulted in 71 articles, while no other studies were identified via going through the available grey literature. After removing the duplicate records, 59 studies were screened afterwards depending on their title and abstract. This process yielded two articles eligible for full-text analysis. The rest of the articles were excluded as their study design, protocol, and methodology aimed to answer a different PICO question to the one that was intended by our study. These two articles [56,57] were ultimately included in the qualitative and quantitative analysis. The flowchart of the article selection process is demonstrated in Figure 1.

The total number of patients in the two RCTs included in this meta-analysis was 654. The total number of patients in the intervention/gemcitabine group was 325, and in the control/placebo group was 329. The median age of patients in both groups in the first study [58] was 66 years, while in the second study [59], it was 65 years in the gemcitabine group and 67 in the placebo group. The total number of male participants was 542 (82.8%). The total number of primary tumors at the time of index TUR across both studies was 448, and the total number of solitary tumors was 407. In the first study [58], patients received either 2 g of gemcitabine in 100 mL of saline or 100 mL of saline alone three hours after TUR and were followed up for a total period of four years. In the second study [59], patients received the same treatment options and had a median follow-up of 23.6 months (range: 0–46 months). The individual characteristics of each included study are mentioned in Table 1.

In the study performed by Messing et al., the recurrence rate for the patients in the gemcitabine group was 35%, while for the placebo group, it was 47%. The calculated hazard ratio with 95% CIs was 0.66 (0.48–0.90) with a *p* < 0.001, indicating a statistically significant difference between the two groups. As part of its secondary analysis, this study also proved a statistically significant difference in the recurrence rate between the gemcitabine and placebo groups for patients diagnosed with low-grade NMIBC (*p* < 0.001), but no such difference was demonstrated in the group of patients with high-grade NMIBC (*p* = 0.38). Moreover, no difference was found in the death rate between the two groups (*p* = 0.12). On the other hand, in the study by Böhle et al., there were 48 recurrences in the gemcitabine group and 46 recurrences in the placebo group, resulting in a hazard ratio of 0.946 (0.64–1.39), which indicates no significant difference between the groups. Subgroup analyses performed by tumor type, number of lesions, and tumor grade also revealed no statistically significant differences.

**Figure 2:**
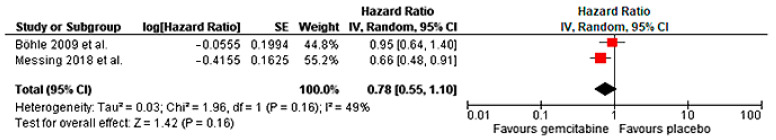
Forest plot showing the outcomes of the meta-analysis.

The meta-analysis of the above-mentioned data showed a pooled hazard ratio of 0.78 (0.55–1.10), indicating that a single adjuvant intravesical dose of gemcitabine does not have a positive outcome on the recurrence rate of NMIBC compared to placebo treatment. The I^2^ test yielded a value of 49%, and the chi^2^ test, a *p*-value of 0.16, indicating that there was homogeneity across the studies included in the meta-analysis. The forest plot of this outcome is shown in Figure 2.

## 4. Discussion

This systematic review and meta-analysis examined the results of two studies on the outcomes of a single adjuvant intravesical instillation of gemcitabine for NMIBC following TUR that were eligible for analysis. We investigated the use of postoperative intravesical instillation of gemcitabin for non-muscle invasive bladder cancer, taking into account the data of these studies. The conclusion was that a single dose of gemcitabine postoperatively does not offer better results in the relapse-free survival of the disease. We have to highlight though the differences between these studies, which may have had a crucial role in this outcome.

Andreas Böhle and his colleagues (2009) conducted a study in which 355 patients were included with histologically confirmed: NMIBC, Ta/T1, G1–3 tumor (Grade 1: 123, Grade 2: 97, Grade 3: 27, grade unknown: 6, not eligible: 80). Out of 355 patients, 78 received a second TUR after the first resection and intravesical therapy. On the other hand, patients in the study performed by Messing et al. did not follow any other treatment after the initial protocol. This second surgery may have changed the postoperative free survival and recurrence of the disease in this group of patients that took part in our research. As there are no data in the literature about the difference between the therapeutical bundle of two TUR and the instillation of gemcitabine or saline, the results from this group of patients have to be taken into consideration.

In the Messing et al. study, 416 patients were included on the basis of tumor appearance that was estimated during office cystoscopy. From this group, 406 were included in the study while 10 did not meet the eligibility criteria. Not all patients from the eligible group that underwent both TUR and instillation stayed at the hospital for 24 h. This means that these patients did not receive any bladder irrigation with saline solution. Patients that took part in the second research protocol were subjected to continuous irrigation with saline for 20 h. Multiple research protocols and meta-analyses have concluded that intravesical irrigation of the urinary bladder with saline for 24 h can decrease the spread of cancer and increase the post-operative free survival of the patient.

It is also worth mentioning that not all chemotherapy agents have been tested using the same parameters, for example, measuring their efficacy with a control group of patients undergoing going continuous intravesical saline irrigation, so we cannot compare the results between other agents and gemcitabine. For example, a study performed by Sugiura Shimpei et al. in 2018 [60] for the Kyoto University included 256 intermediate- and high-risks patients that underwent a single postoperative instillation followed by a BCG regimen, showing a 80.3% progression-free survival rating opposed to 64.5% from the group that only followed the BCG instillation regimen during the follow-up cystoscopy checkups in a period of 5 years. Although the number of patients is small, the results shown were promising.

Gemcitabine is a toxic chemical derivative whose cytotoxic properties can be used for the eradication of cancer cells from the bladder, thus prolonging the overall survival of a patient. Despite the therapeutical factor of gemcitabine, its prolonged stay in the bladder for more than two hours can cause chemical cystitis or other complications. Although the therapeutical window for gemcitabine is one hour, Andreas Böhle’s research team instilled the bladder with gemcitabine for 30–40 min. According to literature, this chemotherapeutical drug should remain in the bladder for at least one hour. This lets us conclude that a large fraction of the patients that took part in this meta-analysis did not follow the time protocol for gemcitabine. Nonetheless, in the two studies included, there were minimal adverse reactions to gemcitabine intravesical infusion, with no grade 4 or 5 adverse effects and similar rates of grade 3 adverse effects in the treatment and placebo groups to the studies of Messing et al. [58] and in Böhle et al. [59], wherein the rates of adverse effects for gemcitabine were 6.6% and 3.7% in the placebo group, respectively. This finding contributes to the findings of Addeo et al. [61], who demonstrated that gemcitabine has a lower toxicity than mitomycin C. Moreover, in the UK, the cost difference between gemcitabine and mitomycin according to the British National Formulary is minimal: a 2 g/200 mL infusion bag of gemcitabine costing GBP 180 and 40 mg of mitomycin powder solvent costing GBP 135 [62,63].

Another point that has to be highlighted is the surgical experience of the surgeons that performed these TURs. In our meta-analysis, the number of patients that were led to the operating room for the resection of their bladder tumor was 738 people. The majority of them were operated by a different practitioner. This logical syllogism adds two more variables that may explain our negative results. The first of those two is that the surgical ability and experience of a surgeon can neither be measured nor compared. In theory, a skilled and experienced surgeon will perform a better resection of a bladder tumor compared to a young and less experienced practitioner. This is not always the case though. In articles where the human variable of a third person (surgeon) plays such a critical role that cannot be calculated, the results can be disputed. The second variable that has to be assessed are the circumstances under which these operations were performed. We have no information about the health units that these operations were carried out. A well-organized hospital as well as a technologically improved unit may offer better therapeutical options for the surgeon, which may have a positive effect in the operating table and the oncological result.

In David Nigel Poller’s et al. report [64], a model for “assessment of errors and diagnostic accuracy in histopathology and cytopathology” was introduced. This study aimed to create a standardized method for the investigation of errors during histopathological practice. The variables included in this method were failure of communication, complacency, lack of knowledge, distractions, lack of teamwork, fatigue, lack of resources, pressure, lack of assertiveness, stress, norms, and lack of awareness. This report helps us to realize the factors that may contribute to a false staging during a histopathological assessment. As mentioned above, the human factor of a single person may have a crucial role in the postoperative free survival of a patient.

Our meta-analysis has some limitations. Although the number of records enrolled through the database search at the beginning was 71, those that took part in quantitative synthesis were two. This means that by having such a small number of patients participating in our record, we cannot be sure about the diversity of our results. On the other hand, this should be the reason for new research teams to help us conclude about the use of gemcitabine after TUR-BT in the general population. Although our meta-analysis showed a pooled hazard ratio of 0.78 leading to a negative result, the differences in medical practice from the two major records that we used may have had a major impact to our results.

Another limitation in our study is that no anatomical or morphological details of the tumor that was resected from these patients was described in the records that we used. This factor can affect subsequent tumor progression.

The third limitation is that there was not a central pathology review. This means that pathologists of varying levels of experience and training reviewed cases of the patients that we enrolled. As mentioned above, this plethora of medical practitioners adds the human factor in the equation.

Finally, this systematic review and meta-analysis was performed without a pre-written and published study protocol. This is another limitation that may have introduced selection bias. Nonetheless, this is considered unlikely as the PICO criteria used to include or exclude studies were very well defined before the process of literature search began.

## 5. Conclusions

This study was a systematic review and meta-analysis on the outcomes of single, post-TUR adjuvant intravesical administration of gemcitabine for the treatment of NMIBC. Our analysis ultimately included two studies with a total number of 654 patients and showed that this therapeutic strategy does not offer a significant benefit to patients with NMIBC in terms of recurrence rate. Nonetheless, on the basis of the generally favorable prognosis of these patients and the various therapeutic agents and schemes available, further studies on the same subject are required to establish the optimal adjuvant therapy in this category of patients to achieve a better clinical outcome.

## Figures and Tables

**Table 1:** Characteristics of each individual study included in the meta-analysis.

Study	Number of Patients	Gemcitabine Group	Placebo Group	Age (Median)	Male Sex	Primary Tumors	Solitary Tumors	Follow-Up
Messing et al. [58]	406	201	205	66	344	256	275	4 years
Böhle et al. [59]	248	124	124	65, gemcitabine group 67, placebo group	198	192	132	23.6 months (median)
Total	654	325	329	N/A	542	448	407	N/A

## Data Availability

The data supporting the findings of this study are available within the article.

## References

[1] Care P. (2017). Bladder cancer: Diagnosis and management of bladder cancer: © NICE (2015) Bladder cancer: Diagnosis and management of bladder cancer. BJU Int..

[2] Kirkali Z., Chan T., Manoharan M., Algaba F., Busch C., Cheng L., Kiemeney L., Kriegmair M., Montironi R., Murphy W.M. (2005). Bladder cancer: Epidemiology, staging and grading, and diagnosis. Urology.

[3] Bhanvadia S.K. (2018). Bladder Cancer Survivorship. Curr. Urol. Rep..

[4] DeGeorge K.C., Holt H.R., Hodges S.C. (2017). Bladder Cancer: Diagnosis and Treatment. Am. Fam. Physician.

[5] Farling K.B. (2017). Bladder cancer: Risk factors, diagnosis, and management. Nurse Pract..

[6] Siegel R.L., Miller K.D., Jemal A. (2015). Cancer statistics, 2015. CA Cancer J. Clin..

[7] Dobruch J., Daneshmand S., Fisch M., Lotan Y., Noon A.P., Resnick M.J., Shariat S.F., Zlotta A.R., Boorjian S.A. (2016). Gender and Bladder Cancer: A Collaborative Review of Etiology, Biology, and Outcomes. Eur. Urol..

[8] Fajkovic H., Halpern J.A., Cha E., Bahadori A., Chromecki T.F., Karakiewicz P.I., Breinl E., Merseburger A.S., Shariat S.F. (2011). Impact of gender on bladder cancer incidence, staging, and prognosis. World J. Urol..

[9] Scosyrev E., Noyes K., Feng C., Messing E. (2008). Sex and racial differences in bladder cancer presentation and mortality in the US. Cancer.

[10] Mungan N.A., Kiemeney L.A.L.M., Van Dijck J.A.A.M., Van Der Poel H.G., Witjes J.A. (2000). Gender differences in stage distribution of bladder cancer. Urology.

[11] Snyder C., Harlan L., Knopf K., Potosky A., Kaplan R.S. (2003). Patterns of Care for the Treatment of Bladder Cancer. J. Urol..

[12] Lyratzopoulos G., Abel G.A., McPhail S., Neal R.D., Rubin G.P. (2013). Gender inequalities in the promptness of diagnosis of bladder and renal cancer after symptomatic presentation: Evidence from secondary analysis of an English primary care audit survey. BMJ Open.

[13] Donsky H., Coyle S., Scosyrev E., Messing E.M. (2014). Sex differences in incidence and mortality of bladder and kidney cancers: National estimates from 49 countries. Urol. Oncol. Semin. Orig. Investig..

[14] Hollenbeck B.K., Dunn R.L., Ye Z., Hollingsworth J.M., Skolarus T.A., Kim S.P., Montie J.E., Lee C.T., Wood D.P., Miller D.C. (2010). Delays in diagnosis and bladder cancer mortality. Cancer.

[15] Chavan S., Bray F., Lortet-Tieulent J., Goodman M., Jemal A. (2013). International Variations in Bladder Cancer Incidence and Mortality. Eur. Urol..

[16] Kluth L.A., Rieken M., Xylinas E., Kent M., Rink M., Rouprêt M., Sharifi N., Jamzadeh A., Kassouf W., Kaushik D. (2013). Gender-specific Differences in Clinicopathologic Outcomes Following Radical Cystectomy: An International Multi-institutional Study of More Than 8000 Patients. Eur. Urol..

[17] Horstmann M., Witthuhn R., Falk M., Stenzl A. (2008). Gender-specific differences in bladder cancer: A retrospective analysis. Gend. Med..

[18] Palou J., Sylvester R.J., Faba O.R., Parada R., Peña J.A., Algaba F., Villavicencio H. (2012). Female gender and carcinoma in situ in the prostatic urethra are prognostic factors for recurrence, progression, and disease-specific mortality in T1G3 bladder cancer patients treated with bacillus Calmette-Guérin. Eur. Urol..

[19] Johnson E.K., Daignault S., Zhang Y., Lee C.T. (2008). Patterns of Hematuria Referral to Urologists: Does a Gender Disparity Exist?. Urology.

[20] Mallin K., David K.A., Carroll P.R., Milowsky M.I., Nanus D.M. (2011). Transitional Cell Carcinoma of the Bladder: Racial and Gender Disparities in Survival (1993 to 2002), Stage and Grade (1993 to 2007). J. Urol..

[21] Soave A., Dahlem R., Hansen J., Weisbach L., Minner S., Engel O., Kluth L., Chun F., Shariat S., Fisch M. (2015). Gender-specific outcomes of bladder cancer patients: A stage-specific analysis in a contemporary, homogenous radical cystectomy cohort. Eur. J. Surg. Oncol..

[22] Cohn J.A., Vekhter B., Lyttle C., Steinberg G., Large M.C. (2013). Sex disparities in diagnosis of bladder cancer after initial presentation with hematuria: A nationwide claims-based investigation. Cancer.

[23] Mitra A.P., Skinner E.C., Schuckman A.K., Quinn D., Dorff T.B., Daneshmand S. (2014). Effect of gender on outcomes following radical cystectomy for urothelial carcinoma of the bladder: A critical analysis of 1,994 patients. Urol. Oncol. Semin. Orig. Investig..

[24] Bassett J.C., Alvarez J., Koyama T., Resnick M., You C., Ni S., Penson D.F., Barocas D.A. (2015). Gender, Race, and Variation in the Evaluation of Microscopic Hematuria Among Medicare Beneficiaries. J. Gen. Intern. Med..

[25] Buteau A., Seideman C.A., Svatek R.S., Youssef R.F., Chakrabarti G., Reed G., Bhat D., Lotan Y. (2014). What is evaluation of hematuria by primary care physicians? Use of electronic medical records to assess practice patterns with intermediate follow-up. Urol. Oncol. Semin. Orig. Investig..

[26] Garg T., Pinheiro L.C., Atoria C.L., Donat S., Weissman J.S., Herr H.W., Elkin E.B. (2014). Gender Disparities in Hematuria Evaluation and Bladder Cancer Diagnosis: A Population Based Analysis. J. Urol..

[27] Borden L.S., Clark P.E., Hall M.C. (2005). Bladder cancer. Curr. Opin. Oncol..

[28] Zeegers M.P., Tan F.E., Dorant E., Brandt P.A.V.D. (2000). The impact of characteristics of cigarette smoking on urinary tract cancer risk: A meta-analysis of epidemiologic studies. Cancer.

[29] Cumberbatch M.G.K., Jubber I., Black P.C., Esperto F., Figueroa J.D., Kamat A.M., Kiemeney L., Lotan Y., Pang K., Silverman D.T. (2018). Epidemiology of Bladder Cancer: A Systematic Review and Contemporary Update of Risk Factors in 2018. Eur. Urol..

[30] Stern M.C., Lin J., Figueroa J.D., Kelsey K.T., Kiltie A., Yuan J.-M., Matullo G., Fletcher T., Benhamou S., Taylor J. (2009). Polymorphisms in DNA Repair Genes, Smoking, and Bladder Cancer Risk: Findings from the International Consortium of Bladder Cancer. Cancer Res..

[31] Afshari M., Janbabaei G., Bahrami M.A., Moosazadeh M. (2017). Opium and bladder cancer: A systematic review and meta-analysis of the odds ratios for opium use and the risk of bladder cancer. PLoS ONE.

[32] Westhoff E., de Oliveira-Neumayer J.M., Aben K.K., Vrieling A., Kiemeney L.A. (2016). Low awareness of risk factors among bladder cancer survivors: New evidence and a literature overview. Eur. J. Cancer.

[33] Crocetto F., Pandolfo S.D., Aveta A., Martino R., Trama F., Caputo V.F., Barone B., Abate M., Sicignano E., Cilio S. (2022). A Comparative Study of the Triglycerides/HDL Ratio and Pseudocholinesterase Levels in Patients with Bladder Cancer. Diagnostics.

[34] Hadkhale K., Martinsen J.I., Weiderpass E., Kjaerheim K., Sparen P., Tryggvadottir L., Lynge E., Pukkala E. (2017). Occupational exposure to solvents and bladder cancer: A population-based case control study in Nordic countries. Int. J. Cancer.

[35] Cumberbatch M.G.K., Cox A., Teare M.D., Catto J. (2015). Contemporary Occupational Carcinogen Exposure and Bladder Cancer. JAMA Oncol..

[36] Liao Y., Huang J.L., Qiu M.X., Ma Z.W. (2014). Impact of serum vitamin D level on risk of bladder cancer: A systemic revew and meta-analysis. Tumor Biol..

[37] Catsburg C.E., Gago-Dominguez M., Yuan J.-M., Castelao J.E., Cortessis V.K., Pike M.C., Stern M.C. (2013). Dietary sources of N-nitroso compounds and bladder cancer risk: Findings from the Los Angeles bladder cancer study. Int. J. Cancer.

[38] Allen N.E., Appleby P.N., Key T.J., Bueno-De-Mesquita H., Ros M.M., Kiemeney L., Tjonneland A., Roswall N., Overvad K., Weikert S. (2013). Macronutrient intake and risk of urothelial cell carcinoma in the European prospective investigation into cancer and nutrition. Int. J. Cancer.

[39] Mendez W.M., Eftim S., Cohen J., Warren I., Cowden J., Lee J.S., Sams R. (2017). Relationships between arsenic concentrations in drinking water and lung and bladder cancer incidence in U.S. counties. J. Expo. Sci. Environ. Epidemiol..

[40] Begum M., Horowitz J., Hossain M.I. (2015). Low-dose risk assessment for arsenic: A meta-analysis approach. Asia-Pac. J. Public Health.

[41] Baris D., Waddell R., Freeman L.E.B., Schwenn M., Colt J.S., Ayotte J.D., Ward M.H., Nuckols J., Schned A., Jackson B. (2016). Elevated Bladder Cancer in Northern New England: The Role of Drinking Water and Arsenic. J. Natl. Cancer Inst..

[42] Desbiolles A., Roudier C., Goria S., Stempfelet M., Kairo C., Quintin C., Monnereau A., Vacquier B., Bidondo M.-L. (2018). Cancer incidence in adults living in the vicinity of nuclear power plants in France, based on data from the French Network of Cancer Registries. Int. J. Cancer.

[43] Finkel M. (2016). Shale gas development and cancer incidence in southwest Pennsylvania. Public Health.

[44] Crocetto F., Zazzo E., Buonerba C., Aveta A., Pandolfo S.D., Barone B., Trama F., Caputo V.F., Scafuri L., Ferro M. (2021). Kaempferol, Myricetin and Fisetin in Prostate and BladderCancer: A Systematic Review of the Literature. Nutrients.

[45] Lenis A.T., Lec P.M., Chamie K. (2020). Bladder cancer a review. JAMA J. Am. Med. Assoc..

[46] Woldu S.L., Bagrodia A., Lotan Y. (2017). Guideline of guidelines: Non-muscle-invasive bladder cancer. Br. J. Urol..

[47] Shen P.-L., Lin M.-E., Hong Y.-K., He X.-J. (2018). Bladder preservation approach versus radical cystectomy for high-grade non-muscle-invasive bladder cancer: A meta-analysis of cohort studies. World J. Surg. Oncol..

[48] Kamat A.M., Bağcıoğlu M., Huri E., Bagcioglu M. (2017). What is new in non-muscle-invasive bladder cancer in 2016?. Turk. Urol. Derg..

[49] Brausi M., Collette L., Kurth K., van der Meijden A.P., Oosterlinck W., Witjes J., Newling D., Bouffioux C., Sylvester R.J. (2002). Variability in the Recurrence Rate at First Follow-up Cystoscopy after TUR in Stage Ta T1 Transitional Cell Carcinoma of the Bladder: A Combined Analysis of Seven EORTC Studies. Eur. Urol..

[50] Van Den Bosch S., Witjes J.A. (2011). Long-term cancer-specific survival in patients with high-risk, non-muscle-invasive bladder cancer and tumour progression: A systematic review. Eur. Urol..

[51] Chang S.S., Boorjian S.A., Chou R., Clark P.E., Daneshmand S., Konety B.R., Pruthi R., Quale D.Z., Ritch C.R., Seigne J.D. (2016). Diagnosis and Treatment of Non-Muscle Invasive Bladder Cancer: AUA/SUO Guideline. J. Urol..

[52] Sylvester R.J., Oosterlinck W., Holmang S., Sydes M., Birtle A., Gudjonsson S., De Nunzio C., Okamura K., Kaasinen E., Solsona E. (2016). Systematic Review and Individual Patient Data Meta-analysis of Randomized Trials Comparing a Single Immediate Instillation of Chemotherapy After Transurethral Resection with Transurethral Resection Alone in Patients with Stage pTa–pT1 Urothelial Carcinoma. Eur. Urol..

[53] Perabo F.G.E., Müller S.C. (2005). New agents in intravesical chemotherapy of superficial bladder cancer. Scand. J. Urol. Nephrol..

[54] Chou R., Selph S., Buckley D.I., Fu R., Griffin J.C., Grusing S., Gore J.L. (2017). Intravesical Therapy for the Treatment of Nonmuscle Invasive Bladder Cancer: A Systematic Review and Meta-Analysis. J. Urol..

[55] Doherty A.P., Trendell-Smith N., Stirling R., Rogers H., Bellringer J. (1999). Perivesical fat necrosis after adjuvant intravesical chemotherapy. Br. J. Urol..

[56] Abdel-Rahman O., Elsayed Z., Elhalawani H. (2018). Gemcitabine-based chemotherapy for advanced biliary tract carcinomas. Cochrane Database Syst. Rev..

[57] Lu J., Xia Q., Lu Y., Liu Z., Zhou P., Hu H., Wang S. (2020). Efficacy of intravesical therapies on the prevention of recurrence and progression of non-muscle-invasive bladder cancer: A systematic review and network meta-analysis. Cancer Med..

[58] Messing E.M., Tangen C.M., Lerner S.P., Sahasrabudhe D.M., Koppie T.M., Wood D.P., Mack P.C., Svatek R.S., Evans C.P., Hafez K.S. (2018). Effect of intravesical instillation of gemcitabine vs saline immediately following resection of suspected low-grade non-muscle-invasive bladder cancer on tumor recurrence SWOG S0337 randomized clinical trial. JAMA J. Am. Med. Assoc..

[59] Böhle A., Leyh H., Frei C., Kühn M., Tschada R., Pottek T., Wagner W., Knispel H.H., von Pokrzywnitzki W., Zorlu F. (2009). Single Postoperative Instillation of Gemcitabine in Patients with Non-muscle-invasive Transitional Cell Carcinoma of the Bladder: A Randomised, Double-blind, Placebo-controlled Phase III Multicentre Study. Eur. Urol..

[60] Sugiura S., Noto N., Koizumi M., Takamoto D., Fujikawa N., Ikeda I. (2018). Post-Operative Immediate Single Instillation of Chemotherapy as Prevention of Recurrence after Transurethral Resection of Intermediate-High Risk Non-Muscle-Invasive Bladder Cancer. Hinyokika Kiyo.

[61] Addeo R., Caraglia M., Bellini S., Abbruzzese A., Vincenzi B., Montella L., Miragliuolo A., Guarrasi R., Lanna M., Cennamo G. (2010). Randomized Phase III Trial on Gemcitabine Versus Mytomicin in Recurrent Superficial Bladder Cancer: Evaluation of Efficacy and Tolerance. J. Clin. Oncol..

[62] MITOMYCIN|Medicinal Forms|BNF Content Published by NICE. https://bnf.nice.org.uk/medicinal-forms/mitomycin.html.

[63] GEMCITABINE|Medicinal Forms|BNF Content Published by NICE. https://bnf.nice.org.uk/medicinal-forms/gemcitabine.html.

[64] Poller D.N., Bongiovanni M., Cochand-Priollet B., Johnson S.J., Perez-Machado M. (2020). A human factor event-based learning assessment tool for assessment of errors and diagnostic accuracy in histopathology and cytopathology. J. Clin. Pathol..

